# Global Prevalence of Adaptive and Prolonged Infections’ Mutations in the Receptor-Binding Domain of the SARS-CoV-2 Spike Protein

**DOI:** 10.3390/v13101974

**Published:** 2021-09-30

**Authors:** Johan Lennerstrand, Navaneethan Palanisamy

**Affiliations:** 1 Department of Medical Sciences, Section of Clinical Microbiology, Uppsala University, 751 85 Uppsala, Sweden; johan.lennerstrand@medsci.uu.se; 2 Chester Medical School, University of Chester, Chester CH2 1BR, UK

**Keywords:** SARS-CoV-2, COVID-19, coronavirus, spike, prevalence

## Abstract

Several vaccines with varying efficacies have been developed and are currently administered globally to minimize the spread of severe acute respiratory syndrome coronavirus 2 (SARS-CoV-2). Despite having an RNA-dependent RNA polymerase with a proofreading activity, new variants of SARS-CoV-2 are on the rise periodically. Some of the mutations in these variants, especially mutations on the spike protein, aid the virus in transmission, infectivity and host immune evasion. Further, these mutations also reduce the effectiveness of some of the current vaccines and monoclonal antibodies (mAbs). In the present study, using the available 984,769 SARS-CoV-2 nucleotide sequences on the NCBI database from the end of 2019 till 28 July 2021, we have estimated the global prevalence of so-called ‘adaptive mutations’ and ‘mutations identified in the prolonged infections’, in the receptor-binding domain (RBD) of the spike (S) protein. Irrespective of the geographical region, in the case of the adaptive mutations, N501Y (48.38%) was found to be the dominant mutation followed by L452R (17.52%), T478K (14.31%), E484K (4.69%), S477N (3.29%), K417T (1.64%), N439K (0.7%) and S494P (0.7%). Other mutations were found to be less prevalent (less than 0.7%). Since the last two months, there has been a massive increase of L452R and T478K mutations (delta variant) in certain areas. In the case of prolonged infections’ mutations (long-term SARS-CoV-2 infections), V483A (0.009%) was found to be dominant followed by Q493R (0.009%), while other mutations were found in less than 0.007% of the studied sequences. The data obtained in this study will aid in the development of better infection control policies, thereby curbing the spread of this virus.

## 1. Introduction

Since its discovery in Wuhan, China, at the end of 2019, severe acute respiratory syndrome coronavirus 2 (SARS-CoV-2: COVID-19) has been wreaking havoc globally, both in terms of human lives and the economy. SARS-CoV-2 belongs to the *Coronaviridae* family, and the members of this family are enveloped, spherical and carry a single-stranded positive-sense RNA (+ssRNA) genome of 27–32 kb [1]. The RNA is capped at the 5′ end, and the 3′ end carries a polyA tail [1]. The genomic RNA codes for two polyproteins, namely ORF1a and ORF1b (via ribosomal frameshifting), are later cleaved by viral proteases into individual functional non-structural proteins [1,2]. These proteins play a role in viral RNA replication and processing [1,2]. The sub-genomic RNA codes for four major structural proteins: spike (S), nucleocapsid (N), membrane (M) and envelope (E) [1]. At the time of writing this manuscript, globally, 199,374,999 SARS-CoV-2 cases have been documented with 4,245,791 deaths (https://www.worldometers.info/coronavirus/, accessed on 3 August 2021).

Coronaviruses are classified into *α*-CoVs, *β*-CoVs and *δ*-CoVs [3,4]. SARS-CoV-2 is categorized under *β*-CoV. Coronaviruses infect both animals and humans. In humans, they can cause both upper and lower respiratory tract infections, and the symptoms can range from a mild cold to bronchitis, pneumonia and SARS [5]. Some coronaviruses that initially existed in the animal populations (enzootic) can jump into humans and can successfully establish diseases in humans [4]. The SARS epidemic between 2002 and 2004 caused by severe acute respiratory syndrome coronavirus 1 (SARS-CoV or SARS-CoV-1), and the periodic Middle East respiratory syndrome (MERS) outbreaks caused by the Middle East respiratory syndrome-related coronavirus (MERS-CoV) since its discovery in 2012, are the best examples of coronaviruses that have crossed the animal–human barrier [6,7]. It is still not clear how these viruses came into the human population in the first place. Some pieces of evidence indicate that these viruses might have jumped from bats to humans via an intermediary host [8,9]. On the genome level, SARS-CoV-2 is 86.85% identical to SARS-CoV-1 and 81.25% identical to MERS-CoV [10].

SARS-CoV-2 spreads mainly via droplets/aerosols from an infected person to a healthy person and via fomites. Further, debates are still ongoing regarding the airborne transmission of SARS-CoV-2 [11,12,13]. After inhalation, in the human body, the virus attaches to membrane-bound angiotensin-converting enzyme 2 (ACE2) using the S protein, and the host serine protease TMPRSS2 primes the S protein for membrane fusion [14]. Once inside the cell, like any other virus, SARS-CoV-2 uses the host’s resources for replication. The pathology is mainly due to the destruction of lung tissues. Currently, there is no effective drug against SARS-CoV-2, although remdesivir and other immunosuppressants are prescribed to reduce the severity of the disease [15,16].

The S protein of coronaviruses elicits a strong T-cell immune response, and since this protein is on the viral surface, especially the S1 subunit, it is a major inducer of virus-neutralizing antibodies [1]. Unlike SARS-CoV-1 and MERS-CoV, approved vaccines have been developed against SARS-CoV-2 and are currently administered to people to reduce the spread of this virus. Many of these vaccines use the S protein as a target [17]. Due to the infidelity of the viral RNA polymerase, unique random template switching during RNA replication and genome plasticity, new variants of SARS-CoV-2 are on the rise [4]. Many of these new variants accumulate mutations on the S protein and several of these mutations aid the virus in transmission, infectivity and host immune evasion. Further, preliminary results suggest that some of these mutations might reduce the effectiveness of some of the current vaccines that target the S protein [18,19].

In addition to vaccines, monoclonal antibody (mAb) therapy for treating SARS-CoV-2 infections has shown promise recently [20]. In addition to use as a treatment, mAbs can also be used as prophylaxis [20,21]. Recently, several neutralizing mAbs have been developed against SARS-CoV-2 and are in different stages of clinical trials. In 2020, the US Food and Drug Administration (FDA) approved the emergency use of neutralizing mAbs for less severe SARS-CoV-2 cases in the USA [20]. Bamlanivimab has been approved to be used either alone or together with etesevimab, and casirivimab with imdevimab [20]. The emergence of new SARS-CoV-2 variants acts as a hurdle to mAb therapy as well as vaccine protection. Similar to the vaccines that target the S protein, some SARS-CoV-2 mutations can also resist neutralization by mAbs currently in clinical trials, to convalescent plasma and plasma from vaccinated individuals [22,23,24,25,26,27,28,29,30,31,32,33,34,35,36,37,38,39]. Table 1 shows mAbs that are in different stages of clinical trials and their effectiveness against different mutations. Therefore, to effectively treat the patients with mAbs and vaccinate high-risk populations, it becomes necessary to know the type of mutation(s)/variant(s) that these patients carry and that are in circulation, respectively. Mutations that aid SARS-CoV-2 in adaptation to the host are called adaptive mutations. Escape mutations, a subgroup of adaptive mutations, help the virus to slip past the host’s immune defenses. Some mutations only emerge/dominate during prolonged infections in immunocompromised patients (prolonged infections’ mutations: long-term COVID-19 infections).

In the present study, using the available nucleotide sequence data on the NCBI COVID-19 database, we estimated the global prevalence of adaptive and prolonged infections’ mutations, in the receptor-binding domain (RBD) of the S protein, i.e., amino acid positions between 333 and 527. While similar prior studies exist [22,46], this study offers the latest update (along with CovMT) and uniquely focuses on the adaptive and prolonged infections’ mutations. Further, in this study, we have shown a simple way of handling a large dataset (roughly 25 GB of sequence data) and acquiring the necessary data within a very short time without the need for massive computing power and advanced programming knowledge. During the last 2 months, a massive increase in the delta variant was reported in the human population (especially in the UK). Thus, we also compared the dynamics of both the adaptive and prolonged infections’ mutations in the global population by initially splitting the whole dataset into two timelines: end of 2019 till 29 May 2021 and 30 May 2021 till 28 July 2021. Further, end of 2019 till 29 May 2021 dataset was split into six timelines and time course of the appearance of the mutations until 29 May 2021 was studied. The data from this study can be used in conjuncture with the coronavirus antiviral and resistance database (CoV-RDB) of Stanford University [45]. We found the prevalence of adaptive mutations in the global population to be quite significant, especially the N501Y, L452R, T478K, E484K and S477N mutations.

## 2. Materials and Methods

A total of 984,769 SARS-CoV-2 nucleotide sequences was retrieved in FASTA format from the NCBI COVID-19 database (https://www.ncbi.nlm.nih.gov/sars-cov-2/) (accessed on 28 July 2021). Based on the release date, the dataset was split into two timelines: end of 2019 till 29 May 2021, and 30 May 2021 till 28 July 2021. For convenience in data handling, the large FASTA files were further split into smaller files using the FASTA Splitter Perl script developed by Kirill Kryukov (http://kirill-kryukov.com/study/tools/fasta-splitter/) (accessed on 28 July 2021). Nucleotide sequences were aligned using the MAFFT v7 alignment program [47]. To do the alignment, a free web server provided by the Osaka University, Japan, was used (https://mafft.cbrc.jp/alignment/server/add_fragments.html?frommanualnov6) (accessed on 28 July 2021). Alignment was performed with default parameters, except for ambiguous sequences, which were not removed during this alignment. SARS-CoV-2 isolate Wuhan-Hu-1 (NCBI accession number: NC_045512.2) was used as the reference for the alignment. The aligned sequences were then processed using AliView (https://ormbunkar.se/aliview/) (accessed on 28 July 2021) [48]. During this stage, sequences that did not contain the region of interest were removed. The nucleotide sequences were then translated into protein sequences using BioEdit v7.2.5, and the protein sequences were exported as an XML file [49]. Table 1 shows the specific mutations studied. These mutations were chosen based on information provided on Stanford University’s CoV-RDB (https://covdb.stanford.edu/page/mutation-viewer/) (accessed on 29 May 2021). Some adaptive mutations, namely Y453F, S477N, T478K, E484K, S494P and N501Y, were also found in long-term COVID-19 infections. Unless stated differently, we considered these mutations as only adaptive. Mutations were counted semi-manually. In the case of countries having more than 50 sequences, Search+ written by Amar Ghosh (https://github.com/amarghosh/searchplus) (accessed on 28 July 2021), a plugin of Notepad++, was used. Compiled Search+ plugin (ready-to-use) for use with Notepad++ 32-bit is provided in the Supplementary Material. Specific tetrapeptide sequences were provided as the input for the search. 

## 3. Results and Discussion

**Figure 1:**
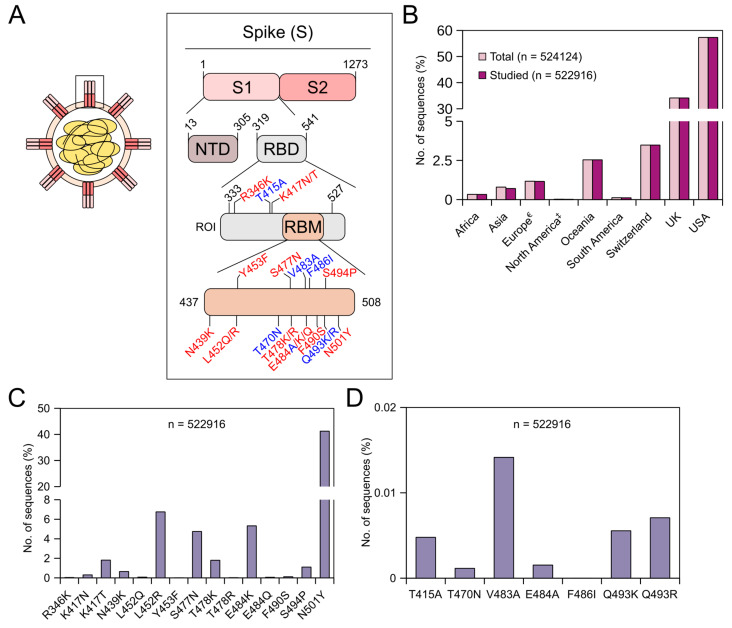
Global prevalence of adaptive and prolonged infections’ mutations in the receptor-binding domain (RBD) of the SARS-CoV-2 spike (S) protein until 29 May 2021. (**A**) SARS-CoV-2 virion (left) and schematics of the S protein (right). The S protein consists of two subunits, namely S1 and S2. The S1 subunit comprises of N-terminal domain (NTD) and receptor-binding domain (RBD). The receptor-binding motif (RBM) is the region within RBD that binds to the angiotensin-converting enzyme 2 (ACE2) receptor. Numbers in black indicate the residue index of the S protein in the SARS-CoV-2 reference genome (NCBI accession number: NC_045512.2). ROI = region of interest. Adaptive mutations are represented in red, while prolonged infections’ mutations are represented in blue. (**B**) Bar graph showing the continent/country-wise distribution of SARS-CoV-2 sequences. For the percentage calculation, the total number of sequences (*n* = 524,124) was used. ^€^ = Europe without Switzerland and the UK. ^‡^ = North America without the USA. (**C**) Bar graph showing the percentages (%) of the respective adaptive mutations. (**D**) Bar graph showing the percentages (%) of the respective prolonged infections’ mutations.

SARS-CoV-2 is rapidly evolving, and countries around the world are struggling to cope with new variants. On 31 May 2021, the WHO named these variants from alpha to kappa (https://www.who.int/en/activities/tracking-SARS-CoV-2-variants/) (accessed on 31 May 2021). These variants have been shown to exhibit reduced susceptibility to some of the mAbs that are under clinical trials, to convalescent plasma and plasma from vaccinated people [22,23,24,25,26,27,28,29,30,31,32,33,34,35,36,37,38,39]. Table 1 shows mutations and their level of resistance to mAbs that are in different stages of clinical trials currently. Further, pieces of evidence suggest that several of these mutations might reduce the effectiveness of some of the vaccines that are given to the public [19,50]. Knowing the prevalence of these mutations will aid in implementing better vaccination strategies and better treatment for patients. Further, as the infection is spread via infected humans to a healthy human, it will aid in postulating better control strategies. Understanding the importance of these mutations, several countries around the globe regularly sequenced the virus collected from their patients and deposited sequence information in some of the public repositories. To this end, we have retrieved 984,769 SARS-CoV-2 nucleotide sequences from the NCBI database for this study. After alignment, nearly 1400 sequences were removed from further analysis, as these sequences lacked the region of interest. Figure 1A shows the residue positions (region of interest) and their corresponding mutation(s) that we studied. To reduce complexity and to make sense of the available mutation data, we considered only specific mutation(s) at specific residue positions, and even in this case, we did not study the prevalence of the entire spectrum of amino acid substitutions at the studied residue positions. Further, we did not study the prevalence of mutations in combinations. The main reason for this is that variants are classified as alpha, beta, etc., based on the presence/absence of signature mutation(s), and not all members within a classification carry the same set of additional mutations. Moreover, as new mutations arise in the human population at regular intervals, the way we infer resistance might change. Thus, we studied stand-alone mutations that have a significant effect on treatment with mAbs. Figure 1B shows the continent/country-wise distribution of the studied SARS-CoV-2 sequences (timeline: end of 2019 till 29 May 2021). From the figure, it can be seen that a major proportion of these sequences come from Switzerland (3.5%), the UK (34.2%) and the USA (57.4%). This is not surprising, as countries with good economies understood the severity of the threat this virus holds and have invested significant resources in sequencing patient samples and became frontrunners in tracking these variants. A report from the *Wall Street Journal* on 30 Jan 2021 stated that the UK became the world leader in sequencing the coronavirus genome as they alerted the world about the identification of the alpha variant, while other countries are lagging (report by Joanna Sugden). Because of this, the prevalence data shown here might also be biased. This could be avoided in the future if more countries around the world come forward in sequencing their patient samples.

### 3.1. Prevalence of Adaptive Mutations Until 29 May 2021

We studied the prevalence of 15 specific adaptive mutations, and the information regarding these mutations was retrieved from the Stanford corona antiviral and resistance database (CoV-RDB) [45]. Irrespective of continents/countries, the prevalence of these adaptive mutations until 29 May 2021 was found to have the following order in the global population; N501Y (41.24%) > L452R (6.75%) > E484K (5.32%) > S477N (4.74%) > K417T (1.82%) > T478K (1.8%) > S494P (1.11%), while the rest of the mutations were found in less than 0.7% of the sequences, with Y453F being the least found (0.01%) (Figure 1C). The Y453F mutation was originally identified in SARS-CoV-2 isolated from minks in Denmark [51]. Although this mutation increases the binding affinity with the ACE2 receptor, the prevalence of this mutation was found to be low in the global human population with the highest prevalence in Denmark (8/14 sequences) [51,52]. Our study also corroborates earlier data. Continent/country-wise prevalence data of adaptive mutations (in %) are provided in Table 2. Raw data for country-wise prevalence are provided in the Supplementary Materials. The K417N/T mutation has been shown to have impaired ACE2 binding [35,53]. The K417N mutation was first identified in South Africa (beta variant), while the K417T mutation was first identified in Brazil (gamma variant) [54,55]. In our study, the highest prevalence of the K417N mutation was found in Finland (2/13 sequences), followed by the Philippines (8/71 sequences). On the other hand, the highest prevalence of the K417T mutation was found in Uruguay (1/10 sequences) followed by Brazil (2/27 sequences). In the case of the N439K mutation, studies have shown enhanced binding to the ACE2 receptor [24,56,57]. This mutation has the highest prevalence in Estonia (3/12 sequences) followed by Austria (25/124 sequences). The L452R mutation was first identified in California, USA (epsilon variant) [58], and as a double mutant with the E484Q mutation in sequences isolated from Maharashtra, India (kappa variant) (BBC News published on 25 March 2021), and also as a double mutant with the T478K mutation in sequences isolated from India (delta variant). In our study, the highest prevalence of L452R was found in Nepal (8/11 sequences), followed by Romania (1/2 sequences). In the UK, the prevalence of this mutation was found in less than 0.07% of the sequences, while in the USA, the prevalence was found to be 11.56%. Like the N439K mutation, the S477N mutation has been shown to exhibit enhanced ACE2 receptor binding [56]. The highest prevalence of this mutation was found in sequences from Australia (10087/13298 sequences), followed by sequences from Hungary (10/36 sequences). The T478K mutation, which is one of the signature mutations in the Indian variant (delta variant), was first identified in sequences isolated from Mexico and California, USA (B.1.1.519 lineage) [59]. In our study, the highest prevalence of the T478K mutation was in sequences from Nepal (7/11 sequences) followed by Morocco (1/14 sequences). The T478R mutation was originally isolated from travelers who travelled within countries in southern Africa [60]. However, in our study, the T478R mutation was identified only in two countries, namely the USA (119/300280 sequences) and the UK (12/178878 sequences). E484K is one of the prime RBM mutations found in many SARS-CoV-2 variants, namely alpha (UK), beta (South Africa), gamma (Brazil), theta (Philippines) and iota (New York, USA) [53,54,55,61,62,63,64,65,66]. Mutations at E484 reduce the binding and neutralization by RBD targeting polyclonal plasma antibodies significantly (>10-fold) [34]. The E484Q mutation, on the other hand, was identified along with the L452R mutation in the kappa variant. The E484K mutation was highly prevalent in Libya (28/34 sequences), followed by South Africa (2/3 sequences), while the E484Q mutation was highly prevalent in Nepal (1/11 sequences) followed by Bahrain (12/269 sequences). The F490S mutation was earlier identified in a few sequences isolated in Peru and Chile [67]. This mutation was found to be highly prevalent in sequences isolated from Peru (26/127 sequences) followed by sequences isolated from the West Bank (6/62 sequences). The S494P mutation was earlier identified in sequences isolated from Santa Cruz County, USA [68]. This mutation has been predicted to have enhanced binding with the ACE2 receptor [69] and was highly prevalent in sequences isolated from Russia (31/304 sequences), followed by sequences isolated from the USA (5167/300280 sequences). Finally, N501Y is another prime RBM mutation found in many SARS-CoV-2 variants, namely alpha, beta, gamma and theta [54,55,65,70,71]. This mutation has been shown to increase the ACE2 binding and enhances the viral replication in the upper respiratory tract [56,72,73,74,75]. More than half of the sequences isolated in the UK until 29 May 2021 were found to carry this mutation, while around 40% of the sequences isolated in the USA during the same period were found to carry this mutation. This mutation was found in greater proportions in sequences isolated from nearly 41 countries. Some of the adaptive mutations that we have studied here have been shown by others to have a moderate to a very high level of resistance (≥100-fold) towards some of the mAbs that are currently either in emergency use in patients or different stages of clinical trials, to convalescent plasma and plasma from vaccinated individuals (Table 1) [22,23,24,25,26,27,28,29,30,31,32,33,34,35,36,37,38,39].

### 3.2. Prevalence of Prolonged Infections’ Mutations Until 29 May 2021

We studied the prevalence of seven specific prolonged infections’ mutations (Table 3). Globally, these mutations were less prevalent in the studied sequences with the V483A mutation having the highest prevalence (0.014%) (Figure 1D). Table 3 shows the continent/country-wise prevalence of prolonged infections’ mutations (in %) in the receptor-binding domain of the SARS-CoV-2 spike protein. Country-wise, V483A mutation was found only in the USA (63/300280 sequences) and the UK (11/178878 sequences). Worldwide, except for the V483A mutation, other mutations were found in <0.008% of the sequences: Q493R (0.007%) > Q493K (0.006%) > T415A (0.005%) > E484A (0.002%) > T470N (0.001%) (Figure 1D). The T415A mutation was found only in sequences from three countries, namely Italy (2/258 sequences), the USA (21/300280 sequences) and the UK (2/178878 sequences). Similar to the V483A mutation, the T470N mutation was also found only in sequences from the USA (4/300280 sequences) and the UK (2/178878 sequences). The E484A mutation was identified only in sequences from Hong Kong (1/339 sequences), the UK (3/178878 sequences) and the USA (4/300280 sequences). The F486I mutation was not found in any of the studied sequences. The Q493K mutation was identified in sequences from four countries, with the highest prevalence in Spain (1/1173 sequences) followed by the UK (13/178878 sequences), Switzerland (1/18234 sequences) and the USA (14/300280 sequences), while the Q493R mutation was identified in sequences from three countries with the highest prevalence in Italy (1/258 sequences) followed by the USA (25/300280 sequences) and the UK (11/178878 sequences). Some of these mutations were either predicted and/or tested to have resistance towards bamlanivimab and etesevimab [76]. The V483A mutation was shown to resist bamlanivimab roughly 48-fold [32], while the E484A mutation was shown to resist C144 mAb greater than 100-fold [37]. Q493K/R mutations, although rare, were found to confer a very high resistance towards bamlanivimab [32], etesevimab [32], casirivimab [23,29,33], C121 [37] and C144 [37].

### 3.3. Prevalence of Adaptive Mutations from 30 May 2021 Till 28 July 2021

**Figure 2:**
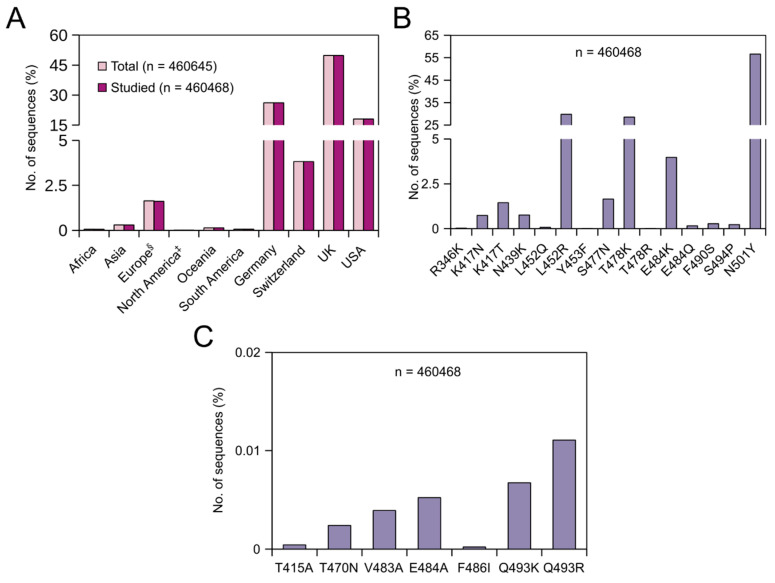
Global prevalence of adaptive and prolonged infections’ mutations in the receptor-binding domain (RBD) of the SARS-CoV-2 spike (S) protein from 30 May 2021 till 28 July 2021. (**A**) Bar graph showing the continent/country-wise distribution of SARS-CoV-2 sequences. For the percentage calculation, the total number of sequences (*n* = 460,645) was used. ^§^ = Europe without Germany, Switzerland and the UK. ^‡^ = North America without the USA. (**B**) Bar graph showing the percentages (%) of the respective adaptive mutations. (**C**) Bar graph showing the percentages (%) of the respective prolonged infections’ mutations.

From 30 May 2021 till 28 July 2021, 460,645 SARS-CoV-2 nucleotide sequences were released in the NCBI database. Of these sequences, 177 sequences were removed from further analysis due to the lack of the region of interest/poor sequence quality. In this dataset, sequences from Germany, Switzerland, the UK and USA constituted about 98% of the total sequences (Figure 2A). Irrespective of continents/countries, the prevalence of these adaptive mutations between 30 May 2021 and 28 July 2021 were found to have the following order in the global population: N501Y (56.61%) > L452R (29.8%) > T478K (28.54%) > E484K (3.97%) > S477N (1.65%) > K417T (1.45%). The rest of the mutations were found in less than 0.8% of the sequences with Y453F being the least found (0.004%) (Figure 2B). During this period, N501Y, L452R and T478K mutations increased by 15.39, 23.06 and 26.75% points, respectively, while E484K, S477N, K417T and S494P mutations decreased by 1.35, 3.09, 0.37 and 0.88% points, respectively. As mentioned earlier, L452R and T478K are signature mutations in the delta variant. Our data confirm that the prevalence of the delta variant has increased considerably in the last two months in the global population. On the continent/country-level, the highest prevalence of the L452R mutation was observed in Asia (57.90%), followed by the UK (49.1%) and the USA (23.34%) (Table 4), and the highest prevalence of the T478K mutation was again observed in Asia (50.59%) followed by the UK (48.63%) and the USA (18.64%) (Table 4). Comparing the two timelines, the prevalence of L452R increased by roughly 56.07, 49.01 and 11.78% points in Asia, the UK and USA, respectively. Similarly, the prevalence of T478K increased roughly by 49.54, 48.62 and 15.57% points in Asia, the UK and USA, respectively. These data indirectly show that the prevalence of the delta variant in the UK has increased tremendously in the last two months. Within Asia, the highest prevalence of the L452R mutation was observed in Uzbekistan (92%) followed by India (75.65%), Bahrain (73.03%) and Myanmar (71.43%) (Supplementary Material). Similarly, the highest prevalence of the T478K mutation was observed in Uzbekistan (76%), followed by Bahrain (73.03%), India (61.36%) and Bangladesh (44.33%) (Supplementary Material). As mentioned before, E484K is one of the prime RBM mutations found in several SARS-CoV-2 variants, and mutations at E484 reduce the binding and neutralization by RBD targeting polyclonal plasma antibodies significantly (>10-fold) [34]. Though a reduction in the prevalence of E484K was observed globally, the prevalence of this mutation has increased by 2.09, 1.06, 0.45 and 5.13% points in Germany, Switzerland, the UK and USA, respectively, in the last 2 months (Figure 1 and Figure 2).

### 3.4. Prevalence of Prolonged Infections’ Mutations from 30 May 2021 Till 28 July 2021

Similar to the previous timeline, globally, these mutations were less prevalent. However, unlike the previous timeline, during this period, Q493R (0.011%) was found to be the dominant mutation followed by Q493K (0.007%), E484A (0.005%), V483A (0.004%), T470N (0.002%), T415A and F486I (Figure 2C). During this period, the prevalence of Q493R, Q493K, E484A and T470N increased by 0.004, 0.001, 0.003 and 0.001% points, respectively, while the prevalence of V483A and T415A decreased by 0.01 and 0.005% points, respectively. As mentioned before, the Q493R mutation alone confers significant resistance towards bamlanivimab, etesevimab, casirivimab and C144 [32,37,42]. On the continent/country level, this mutation was found in only sequences from three countries: the USA (24/83122 sequences), the UK (23/229321 sequences) and Germany (4/120429 sequences) (Table 5). Q493K and E484A mutations were found in only sequences from Switzerland, the UK, USA and Germany. Of these four countries, both these mutations were found to be highly prevalent in sequences isolated from Switzerland (Table 5). Unlike the previous timeline, V483A was additionally identified in sequences from Egypt and Germany. It is worth noting that 3 out of 65 sequences from Egypt carried this mutation (Supplementary Material). Until 29 May 2021, the T470N mutation was identified only in sequences from the UK and USA. During the last two months, this mutation was confined within Europe, especially in Estonia, Germany, Switzerland and the UK. In Estonia, 5 out of 1635 sequences carried this mutation (Supplementary Material). During the same period, on the other hand, the T415A mutation was confined within the USA (Table 5), and the prevalence almost doubled. Finally, until 29 May 2021, the F486I mutation was not identified in any of the sequences. Between 30 May 2021 and 28 July 2021, this mutation was identified in one of the 83,122 studied sequences from the USA.

### 3.5. Time Course of the Appearance of the Mutations Until 29 May 2021

To study the time course of the appearance of the mutations, we split the data into six timelines, with each timeline covering about 3 months except the last timeline, which covered approximately 4 months: Timeline 1 (1 November 2019 till 31 January 2020: T1), Timeline 2 (1 February 2020 till 30 April 2020: T2), Timeline 3 (1 May 2020 till 31 July 2020: T3), Timeline 4 (1 August 2020 till 31 October 2020: T4), Timeline 5 (1 November 2020 till 31 January 2021: T5) and Timeline 6 (1 February 2021 till 29 May 2021: T6). T1, T2, T3, T4, T5 and T6 had the following respective sequence distributions: 0.006%, 0.32%, 2.07%, 4.68%, 3.81% and 90.13%. This shows that a similar number of SARS-CoV-2 sequences were released by the NCBI from 1 February 2021 to 29 May 2021 and from 30 May 2021 to 28 July 2021. In T1, none of the sequences carried any adaptive and prolonged infections’ mutations. In T2, nearly 0.76% of sequences from the USA carried the V483A mutation. Country-wise raw data on the prevalence of SARS-CoV-2 receptor-binding domain (RBD) mutations concerning different timelines is provided in the Supplementary Material. No other mutation was observed in T2. In T3, mutations N439K, Y453F, S477N, V483A, E484K, E484Q, S494P and N501Y were observed. While N439K, Y453F, V483A, E484K, S494P and N501Y mutations were only observed in sequences from the USA (0.015%), Netherlands (33.33%), USA (0.21%), USA (0.01%), USA (0.04%) and Australia (0.99%), respectively, S477N and E484Q were identified in more than one country with the highest prevalence in Lebanon (25%) and India (0.44%), respectively. In T4, apart from the mutations identified in T3, mutations R346K, L452Q, L452R and T478K were observed. While R346K and L452R mutations were observed only in sequences from Iraq with a prevalence of 7.41% and 1.85%, respectively, the other two mutations were observed only in sequences from the USA, with each having a prevalence of approximately 0.009%. In T5, except for the L452Q mutation, all other mutations discussed above were observed. Additionally, mutations K417N, K417T, T470N, T478R, E484A, F490S and Q493K were observed. All these mutations were observed only in sequences from the USA except for the K417T mutation, which was also found in a sequence from Italy. The prevalences of these mutations were quite low (≤0.04%). Finally, mutations T415A and Q493R were observed in T6 apart from the other mutations discussed above. Both these mutations were identified only in sequences from the USA, UK and Italy. It is worth noting that both these mutations were found to be highly prevalent in sequences from Italy compared to those from the USA and UK.

In conclusion, globally, we found a high prevalence of N501Y mutation in the RBD of the SARS-CoV-2 S protein using the available sequence data from the NCBI database up to 28 July 2021. Further, we observed a considerable prevalence of other adaptive mutations in the RBD, namely L452R, T478K, E484K, S477N, K417T, N439K and S494P. Prolonged infections’ mutations were observed only in a few sequences from a few countries. A few drawbacks in our study, like any other epidemiological study, are the available number of sequences from each country, the context to which the sequencing was done in the first place, etc. Because of these reasons, the data presented here might be skewed and should be used with caution. Further, we did not use the sequences from the GISAID (https://www.gisaid.org/) (accessed on 3 August 2021) database, which contained 2,568,717 sequences at the time of writing this manuscript, as one needs prior permission to use the data. Thus, we used only sequences from the NCBI database as they are readily accessible by anyone without any registration and/or signing any agreement. Moreover, the GISAID database has inbuilt visualization software using which variants (mutations) can be analyzed. In addition, many online visualization tools (e.g., CovMT) use data from the GISAID database. To our knowledge, no such tools use sequence data deposited in the NCBI database. To analyze large sequence data, in many cases, specialized computational skills/hardware requirements are needed. This motivated us to use the data from the NCBI database and develop a methodology. It should be noted that not all SARS-CoV-2 sequences are deposited in the NCBI and GISAID databases. Anyone interested in studying the prevalence of mutations (variants of concern, variants of interest and variants under monitoring) from sequences isolated locally or regionally can follow our methodology, and our methodology is not limited only to the SARS-CoV-2 sequences. Like the UK, USA, Germany and Switzerland, many countries should actively sequence as many SARS-CoV-2 sequences as possible from patients and deposit them in the public repository. This will aid in a better understanding of the viral evolution in the human population. As more sequence information gets deposited in the public database regularly, we might/will observe a change in the prevalence landscape shortly, as we have observed for the delta variant. Thus, it is necessary to do this type of study at regular intervals to keep ourselves updated about the latest dominant mutation(s), both globally and regionally (or locally), and thereby we can evolve our control strategies and put a full stop to the spread of this devastating viral infection.

## Figures and Tables

**Table 1:** List of adaptive and prolonged infections’ mutations in the RBD of the SARS-CoV-2 S protein studied and their resistance to mAbs that are in emergency use and clinical trials ^⁑^.

Type	Mutation	Resistance to mAbs ^#^ (Fold Change Compared to SARS-CoV-2 Wuhan-Hu-1 Isolate)
Adaptive	R346K	C135 (≥100-fold) ^PVH, PVV^ [37]
	K417N	casirivimab (43-fold) ^PVM^ [40], (5.7-fold) ^PVH^ [36], (7-fold) ^PVV^ [32]
	K417T	data not available
	N439K	C135 (≥100-fold) ^PVH^ [37], (33.1-fold) ^PVV^ [37]; imdevimab (5-fold) ^PVV^ [41], (>100-fold) ^PVV^ [32]
	L452Q	data not available
	L452R	bamlanivimab (≥100-fold) ^PVV^ [32]; bamlanivimab/etesevimab (7.4-fold) ^PVV^ [32]
	Y453F	casirivimab (≥100-fold) ^PVV^ [29,32], (>25.6-fold) ^PVV^ [41]; casirivimab/imdevimab (3.5-fold) ^PVV^ [29]
	S477N	no resistance to current mAbs
	T478K	no resistance to current mAbs
	T478R	data not available
	E484K	bamlanivimab (≥100-fold) ^PVV^ [31,32]; bamlanivimab/etesevimab (17-fold) ^PVV^ [32]; C135 (3.5-fold) ^PVH^ [37]; C144 (≥100-fold) ^PVH, PVM^ [37,40]; casirivimab (20.2-fold) ^PVV^ [29], (55-fold) ^PVM^ [40], (4.4-fold) ^PVV^ [41], (25-fold) ^PVV^ [32]; casirivimab/imdevimab (4-fold) ^PVM^ [40]; etesevimab (4.4-fold) ^PVV^ [31]
	E484Q	bamlanivimab (≥100-fold) ^PVV^ [31,32]; bamlanivimab/etesevimab (22-fold) ^PVV^ [32]; C144 (≥100-fold) ^PVH, PVV^ [37,42]; casirivimab (19-fold) ^PVV^ [32], (63.3-fold) ^PVH^ [43]; casirivimab/imdevimab (4.5-fold) ^PVH^ [43]
	F490S	bamlanivimab (≥100-fold) ^PVV^ [31,32]; C144 (4.9-fold) ^PVH^ [37]
	S494P	bamlanivimab (>100-fold) ^PVV^ [31], (>71-fold) ^PVV^ [32]; C144 (>100-fold) ^PVH^ [42], (60.8-fold) ^PVH^ [37], (17-fold) ^PVV^ [37]; casirivimab (5-fold) ^PVV^ [32]
	N501Y	etesevimab (20-fold) ^PVM^ [44], (5-fold) ^PVV^ [32]; imdevimab (15.6-fold) ^PVM^ [44]
Prolonged infections	T415A	data not available
	T470N	data not available
	V483A	bamlanivimab (48-fold) ^PVV^ [32]
	E484A	C144 (≥100-fold) ^PVH, PVV^ [37,42]
	F486I	data not available
	Q493K	C144 (≥100-fold) ^PVH, PVV^ [37]; casirivimab (≥100-fold) ^PVV^ [29,32], (>25.6-fold) ^PVV^ [41]
	Q493R	bamlanivimab (≥100-fold) ^PVV^ [32]; bamlanivimab/etesevimab (≥100-fold) ^PVV^ [32]; casirivimab (70-fold) ^PVV^ [32]; C144 (≥100-fold) ^PVH, PVV^ [37,42]; imdevimab (5-fold) ^PVV^ [32]

^⁑^ Residue positions and resistance data were taken from Stanford University’s coronavirus antiviral and resistance database (CoV-RDB) using the following link: https://covdb.stanford.edu/ (accessed on 29 May 2021) [45]. ^#^ Resistance ≤3.4-fold is not shown here. ^PVH^ = pseudovirus (HIV), ^PVM^ = pseudovirus (MLV), ^PVV^ = pseudovirus (VSV).

**Table 2:** Continent/country-wise prevalence of adaptive mutations in the receptor-binding domain of the SARS-CoV-2 spike protein until 29 May 2021.

Continent/Country	No. of Studied Sequences	Mutation (%)														
		R346K	K417N	K417T	N439K	L452Q	L452R	Y453F	S477N	T478K	T478R	E484K	E484Q	F490S	S494P	N501Y
**Africa**	1728		0.347		0.289		5.845	0.058	1.678	0.058		4.456	0.174		0.058	9.028
Asia	3703	0.135	1.242	0.351	0.270	0.027	1.836		0.513	1.053		1.782	0.702	0.189		7.399
Europe ^€^	6092	0.033	0.509	0.312	1.346		0.575	0.230	5.368	0.066		1.625	0.033		0.657	26.133
Switzerland	18,234	0.362	0.516	0.126	4.442	0.005	1.475	0.016	21.213	0.801		0.856	0.038	0.033	0.104	17.906
UK	178,878	0.003	0.084	0.004	1.348	0.002	0.070	0.004	1.094	0.010	0.007	0.214	0.013	0.051	0.323	51.055
North America ^‡^	99								2.020			9.091				1.010
USA	300,280	0.041	0.424	3.147	0.034	0.119	11.556	0.010	2.836	3.067	0.040	9.005	0.089	0.162	1.721	39.631
Oceania	13,299								75.848						0.008	0.278
South America	603			0.663		4.312						1.990		4.312		0.995

^€^ = Europe excluding Switzerland and the UK; ^‡^ = North America excluding the USA; empty cell = 4 or more decimal places or 0%.

**Table 3:** Continent/country-wise prevalence of prolonged infections’ mutations in the receptor-binding domain of the SARS-CoV-2 spike protein until 29 May 2021.

Continent/Country	No. of Studied Sequences	Mutation (%)						
		T415A	T470N	V483A	E484A	F486I	Q493K	Q493R
**Africa**	1728							
Asia	3703				0.027			
Europe ^€^	6092	0.033					0.016	0.016
Switzerland	18,234						0.005	
UK	178,878	0.001	0.001	0.006	0.002		0.007	0.006
North America ^‡^	99							
USA	300,280	0.007	0.001	0.021	0.001		0.005	0.008
Oceania	13,299							
South America	603							

^€^ = Europe excluding Switzerland and the UK; ^‡^ = North America excluding the USA; empty cell = 4 or more decimal places or 0%.

**Table 4:** Continent/country-wise prevalence of adaptive mutations in the receptor-binding domain of the SARS-CoV-2 Scheme 30 May 2021 till 28 July 2021.

Continent/ Country	No. of Studied Sequences	Mutation (%)														
		R346K	K417N	K417T	N439K	L452Q	L452R	Y453F	S477N	T478K	T478R	E484K	E484Q	F490S	S494P	N501Y
**Africa**	271		11.808				11.439			1.107		20.664	0.369	0.738	3.321	49.815
Asia	1354		7.090	0.148	0.074		57.903		0.148	50.591	0.074	6.499	6.721	0.074		23.560
Europe ^§^	7400	0.014	0.311	0.041	3.608	0.027	0.122		1.041	0.027		0.554	0.014	0.041	0.027	17.270
Germany	120,429	0.033	1.476	0.453	2.148	0.121	3.310	0.002	2.352	2.819	0.011	3.582	0.134	0.542	0.174	79.022
Switzerland	17,578	0.233	0.688	0.484	2.463	0.017	2.890		9.148	2.020	0.006	1.911	0.091	0.307	0.165	58.095
UK	229,321	0.007	0.256	0.065	0.090	0.003	49.075		0.025	48.633		0.666	0.171	0.112	0.054	48.698
North America ^‡^	66						3.030			1.515		6.061				1.515
USA	83,122	0.066	0.929	6.950	0.011	0.217	23.336	0.018	3.621	18.640	0.017	14.132	0.109	0.393	0.829	50.363
Oceania	634								0.946							
South America	293			37.201	0.341	1.024						62.457		1.024	0.341	39.249

^§^ = Europe excluding Germany, Switzerland and the UK; ^‡^ = North America excluding the USA; empty cell = 4 or more decimal places or 0%. Prevalence of prolonged infections’ mutations from 30 May 2021 till 28 July 2021.

**Table 5:** Continent/country-wise prevalence of prolonged infections’ mutations in the receptor-binding domain of the SARS-CoV-2 spike protein from 30 May 2021 till 28 July 2021.

Continent/ Country	No. of Studied Sequences	Mutation (%)						
		T415A	T470N	V483A	E484A	F486I	Q493K	Q493R
**Africa**	271			1.107				
Asia	1354							
Europe ^§^	7400		0.068					
Germany	120,429		0.001	0.004	0.002		0.002	0.003
Switzerland	17,578		0.006		0.057		0.011	
UK	229,321		0.002	0.001	0.003		0.010	0.010
North America ^‡^	66							
USA	83,122	0.002		0.010	0.006	0.001	0.006	0.029
Oceania	634							
South America	293							

^§^ = Europe excluding Germany, Switzerland and the UK; ^‡^ = North America excluding the USA; empty cell = 4 or more decimal places or 0%.

## Supplementary Materials

The following are available online at https://www.mdpi.com/article/10.3390/v13101974/s1, Country-wise raw data on the prevalence of SARS-CoV-2 receptor-binding domain (RBD) mutations: 2019 till 29 May 2021, 30 May 2021 till 28 July 2021 and different time lines (T1–T6) are provided as Excel files. Compiled Search+ plugin (ready-to-use) for use with Notepad++ 32-bit is also provided.
